# Browning of Boreal Freshwaters Coupled to Carbon-Iron Interactions along the Aquatic Continuum

**DOI:** 10.1371/journal.pone.0088104

**Published:** 2014-02-05

**Authors:** Gesa A. Weyhenmeyer, Yves T. Prairie, Lars J. Tranvik

**Affiliations:** 1 Department of Ecology and Genetics/Limnology, Uppsala University, Uppsala, Sweden; 2 Département des Sciences Biologiques, Université du Québec à Montréal, Montreal, Quebec, Canada; University of Yamanashi, Japan

## Abstract

The color of freshwaters, often measured as absorbance, influences a number of ecosystem services including biodiversity, fish production, and drinking water quality. Many countries have recently reported on increasing trends of water color in freshwaters, for which drivers are still not fully understood. We show here with more than 58000 water samples from the boreal and hemiboreal region of Sweden and Canada that absorbance of filtered water (a_420_) co-varied with dissolved organic carbon (DOC) concentrations (*R^2^* = 0.85, *P*<0.0001), but that a_420_ relative to DOC is increased by the presence of iron (Fe). We found that concentrations of Fe significantly declined with increasing water retention in the landscape, resulting in significantly lower Fe concentrations in lakes compared to running waters. The Fe loss along the aquatic continuum corresponded to a proportional loss in a_420_, suggesting a tight biogeochemical coupling between colored dissolved organic matter and Fe. Since water is being flushed at increasing rates due to enhanced runoff in the studied regions, diminished loss of Fe along the aquatic continuum may be one reason for observed trends in a_420_, and in particular in a_420_/DOC increases. If trends of increased Fe concentrations in freshwaters continue, water color will further increase with various effects on ecosystem services and biogeochemical cycles.

## Introduction

During the last decade there have been several reports of increasing water color in the Northern Hemisphere, first reviewed by [1] and later confirmed by various other studies, e.g. [2]–[4]. The trends have frequently been attributed to increasing dissolved organic carbon (DOC) concentrations, resulting from changes in both climate and atmospheric deposition [5]. Recently, also the importance of increasing iron (Fe) concentrations have been pointed out as a possible driver for water color increases [6], [7]. Fe and DOC are not independent from each other since Fe can form stable complexes with DOC [8]. Such complexes turn waters into a dark, brownish color, with a pronounced effect on absorbance measures [6], [9], [10].

In a recent laboratory study [11], it has been shown that addition of Fe to DOC solutions resulted in a linear increase in the light absorption at a wavelength of 410 nm. This linear increase continued until a maximum Fe binding capacity to dissolved organic matter was reached and Fe precipitated _ENREF_33[11]. Thus, apart from a DOC effect on absorbance, Fe has an additive effect on absorbance until dissolved organic matter becomes saturated. How frequent an additive Fe effect on absorbance in natural waters occurs is not known yet. We therefore used data on Fe, DOC and absorbance at 420 nm (a_420_) from more than 58000 water samples of the Swedish and Canadian boreal and hemiboreal region and analyzed how Fe contributes to a_420_ relative to DOC. We hypothesized that Fe concentrations are strongly positively related to a_420_/DOC ratios. Since a_420_/DOC ratios have previously been shown to decrease along the aquatic continuum [12], [13] we further hypothesized that decreases in a_420_/DOC are related to Fe concentration decreases along the aquatic continuum. Along the aquatic continuum we had data from small headwater streams, non-headwater streams, lakes, large lakes, and river mouths.

## Material and Methods

### Databases

In this study we used 58888 Swedish water samples from 6339 lakes, including Sweden's three largest lakes Vänern, Vättern and Mälaren, 209 streams, including 11 small headwater stream sites and 52 river mouths. The water systems are distributed all over Sweden, and represent waters of the boreal and hemiboreal region. Most of the 6339 lakes are small (median lake area: 0.16 km^2^), shallow (median mean lake depth: 3.2 m) and nutrient poor (median total phosphorus concentration: 11 µg L^−1^) lakes with a median pH of 6.7. All data are from a water depth of 0.5 m and have been sampled and analyzed by the laboratory of the Department of Aquatic Sciences and Assessment at the Swedish University of Agricultural Sciences according to standard limnological methods. A detailed method description and all data can freely be downloaded at http://webstar.vatten.slu.se/db.html.

The data have been derived during the past 30 years. Most water systems were sampled more than once, and 66 water systems had monthly Fe, DOC and absorbance data available, in lakes at least during the ice-free season (usually May to October) since 1996. These 66 water systems comprised 21 lakes, 11 streams and 34 river mouths. Apart from time series we had 7196 water samples that originated from large-scale lake inventories during autumns between 2000 and 2012, taken at 0.5 m when the water column was mixed at water temperatures around 4°C. More information on these large-scale lake inventories can be found at http://webstar.vatten.slu.se/db.html.

In addition to the Swedish data we also used a dataset (247 water samples) of 30 Canadian boreal lakes to verify our results. The Canadian data as well as methods are available in [14].

Finally, we also used a database on meteorological variables, available at http://www.smhi.se. In this database, named climate indicators, 10-year running means of annual precipitation, air temperature and growing season length based on data from meteorological stations across entire Sweden are available.

### Variables

For all 58888 water samples we had data on total iron, total organic carbon and on absorbance of 0.45 µm filtered water at 420 nm in a 5-cm cuvette (AbsF_420nm/5cm_). Although iron and organic carbon were measured as total concentrations they are in this study considered as dissolved. This assumption is based on previous investigations where it was shown that in Swedish boreal waters particulate organic carbon is negligible as it only accounts for less than 5% of the total organic carbon [15]. To further strengthen the assumption of negligible particulate matter influence on iron and organic carbon measurements in Swedish boreal waters we compared absorbance of filtered and unfiltered water at 420 nm, which we had available for 46787 out of the 58888 water samples. We received a very good correspondence (*R^2^* = 0.83, *P*<0.0001, *n* = 46787) and a slope of 1.2. The slope exceeded 1 at lowest iron and organic carbon concentrations but approached 1.0 at high concentrations of both iron and organic carbon. Thus, in Swedish boreal waters high iron and organic carbon concentrations occurred when particulate matter in the water samples was absent. Consequently, we considered the influence of particulate matter on iron and organic carbon measurements negligible and used the abbreviations Fe and DOC throughout the text. We, however, chose the absorbance ratio between unfiltered and filtered water at 420 nm as a variable to account for variations in particulate matter in our water samples.

We converted AbsF_420nm/5cm_ data to the Napierian absorption coefficient as recommended by [16], according to:
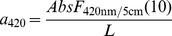
(1)where a_420_ is the Napierian coefficient in m^−1^, AbsF_420nm/5cm_ is the measured absorbance of filtered water, ln(10) is the natural logarithm of 10 and L is the optical path-length in m. For the Canadian data we had to use an additional transformation as absorbance of filtered water was measured at 440 nm in a 10-cm cuvette (AbsF_440nm/10cm_). For the transformation we used the following equation according to [17]:
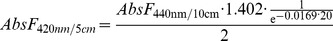
(2)


Apart from Fe, DOC and a_420_ we had, for 5837 waters comprising 5664 lakes, 6 small headwater streams and 167 non- headwater streams, additional data on 18 water physical and chemical variables as well as 21 GIS derived catchment variables. The variables, that were also available for 66 water systems with complete time series, included water temperature, pH, alkalinity, conductivity, calcium, magnesium, sodium, potassium, chloride, sulfate, ammonium-nitrogen, nitrate-nitrogen, total nitrogen, total phosphorus, reactive silica, manganese, size of catchment area, site-specific altitude, site-specific annual precipitation (average 1961–1990), site-specific annual mean air temperature (average 1961–1990), site-specific growing season length (average 1961–1990), site-specific annual global radiation (average 1961–1990), and percentage of grass, other cultured land, coniferous forest, coniferous forest on wetland, pasture, mixed forest, mixed forest on wetland, exploited land, clear-cut, deciduous, urban, agriculture, open wetland, other vegetation and lake surface cover in the catchment area. In this study we used the percentage of lake surface cover in the catchment area (% Water) as a proxy for water retention in the landscape. We justify this approach by a significant relationship between calculated water retention in the Swedish landscape and % Water (*R* ^2^ = 0.27, *P*<0.0001, *n* = 1419 based on data published by [12]).

### Statistics

All statistical tests, run in JMP, version 10.0, considered the non-normal distribution of the data material by using log-transformations for linear relationships and by applying non-parametric tests. Tests we used were: A) Simple linear relationships. When the data were non-normally distributed according to a Shapiro-Wilk test we log-transformed the data which in all cases was successful in receiving normally distributed data. B) Partial least squares regression models (PLS). The PLS allowed to predict a_420_ and a_420_/DOC by a variety of water and catchment variables. We chose PLS because of the method's insensitivity to *X*-variable's interdependency and the insensitivity to deviations from normality [18]_ENREF_2. PLS is commonly used to find fundamental relations between two matrices (*X* and *Y*) where the variance in *X* is taken to explain the variance in *Y*. In PLS, *X*-variables are ranked according to their relevance in explaining *Y*, commonly and also in this study expressed as VIP-values [18]. The higher the VIP values are the higher is the contribution of an *X*-variable to the model performance. VIP-values exceeding 1 are considered as important *X*-variables. In this study, we restricted our discussions to very important *X*-variables, i.e. variables that had VIP values exceeding 1.8. The PLS modeling approach was applied for median values of 5837 water systems for which a variety of water and catchment variables were available. C) Standard least squares models. Standard least squares models are special cases of PLS where single *Y*-variables are predicted. The models allow an immediate graphical comparison between predicted and measured values. We used these models for predictions of a_420_ and a_420_/DOC with DOC, Fe and a_420unfiltered_/a_420filtered_ as input variables. D) Wilcoxon-test. The Wilcoxon test is a non-parametric test for group comparison. We applied this test for a comparison of variables between headwater streams, non-headwater streams, lakes, large lakes and river mouths. E) Mann-Kendall trend analyses. The non-parametric Mann-Kendall test [19] gives a measure whether long-term changes of a variable are significant (*P*<0.05) or not (*P*≥0.05). We applied Mann-Kendall tests on annual mean values of variables that came out as significant in the PLS modelling approach (see test B described above). For the analyses we used the data from the 66 water systems with complete monthly time series. F) De-trending methods. To assess a Fe-independent DOC effect on a_420_ we de-trended a_420_ by Fe, i.e. we used the residuals of a linear relationship between log Fe and log a_420_ and related these residuals to DOC concentrations. Likewise we assessed the DOC-independent Fe influence on a_420_ by relating the residuals of a log DOC- log a_420_ relationship to Fe. For model results we restricted ourselves to report on *R^2^* values since they were equal to *R^2^* adjusted values when we considered the first two decimals.

## Results

### Variations in a_420_ and main drivers

The median a_420_ of 58888 Swedish water samples was 5.58 m^−1^ (5^th^ percentile: 0.83 m^−1^ and 95^th^ percentile: 22.15 m^−1^). Highest a_420_ values were observed in streams (median: 6.40 m^−1^) and lowest in large lakes (median: 1.86 m^−1^). Using partial least squares analysis to predict variations in a_420_ across 5837 waters for which we had complete catchment and water physico-chemical data available (21 and 18 variables, respectively; see methods) we found that DOC, Fe, pH and Si were most influential in explaining a_420_ variations (highest VIP values, all exceeding 1.8, in a partial least squares analysis). From the catchment variables the percentage of coniferous forest and the percentage of lake surface cover in the catchment came out as most influential for a_420_ variations (VIP value 1.6 and 1.3, respectively). The strongest relationship of a_420_ to a single variable was achieved for DOC (linear relationship on log-transformed data: *R^2^* = 0.82, *P*<0.0001, *n* = 5837). Also Fe was highly significantly related to a_420_ (linear relationship on log-transformed data: *R^2^* = 0.73, *P*<0.0001, *n* = 5837). Considering both DOC and Fe as input variables in a standard least squares model we were able to explain 89% of a_420_ variations across the 5837 water systems, and 86% when we used all available DOC and Fe data from 58888 water samples. For the model performance, both DOC and Fe made significant contributions (*P*<0.0001, *n* = 58888). When the influence of Fe was separated from the DOC signal on a_420_ by linear de-trending (see method F in method part) we found that DOC could only explain 38% of a_420_ from which the Fe signal had been removed (linear relationship: *R^2^* = 0.38, *P*<0.0001, *n* = 58888). Likewise the Fe contribution to a_420_ from which the DOC signal had been removed was reduced to 25% (*P*<0.0001, *n* = 58888).

**Figure 1:**
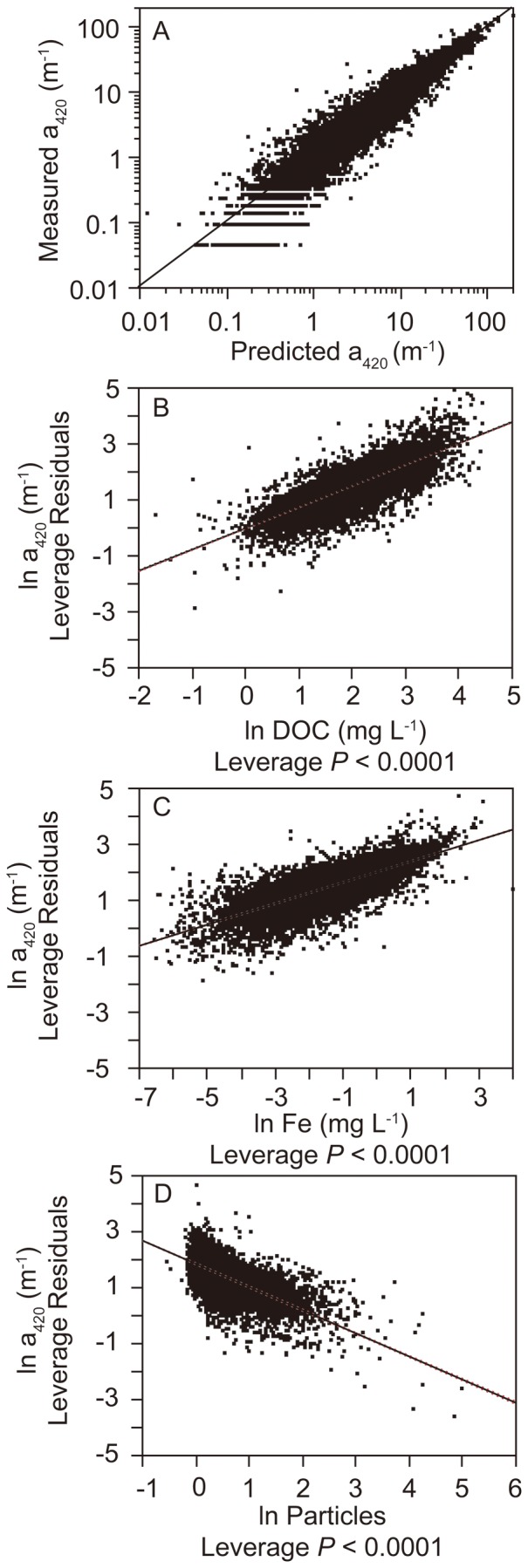
Prediction of absorbance (a_420_) by dissolved organic carbon (DOC), iron (Fe) and particulate matter (particles) for 46787 Swedish water samples. Particulate matter was assessed by the absorbance ratio between unfiltered and filtered water (see methods). 92% of the a_420_ variations could be explained by the simple standard least squares model (panel A; a_420_ = e^(0.73+0.76·ln*DOC*+0.38·ln*Fe*−0.83·ln*Particles*)^) where all three input variables made a significant contribution to the model performance, here shown by model leverage plots (panels B–D). Removing the input variable particles from the model, the model performance decreased to *R* ^2^ = 0.85, *P*<0.0001, *n* = 46787. Using only DOC as input variable the model performance was *R* ^2^ = 0.73, *P*<0.0001, *n* = 46787.

Examining the residuals of the standard least squares a_420_ model with DOC and Fe as input variables we found them highly negatively related to the amount of particulate matter in the water, here defined as the absorbance ratio between unfiltered and filtered water at 420 nm (linear relationship: *R^2^* = 0.47, *P*<0.0001, *n* = 46787). Adding the influence of particulate matter as input variable in the a_420_ model we were able to predict as much as 92% of a_420_ variations across various temporal and spatial scales (Fig. 1a). All three input variables had a highly significant influence on the model performance (Fig. 1b–c). Without particulate matter the model performance decreased to *R* ^2^ = 0.85, *P*<0.0001, *n* = 46787.

### Variations in a_420_/DOC and main drivers

**Figure 2:**
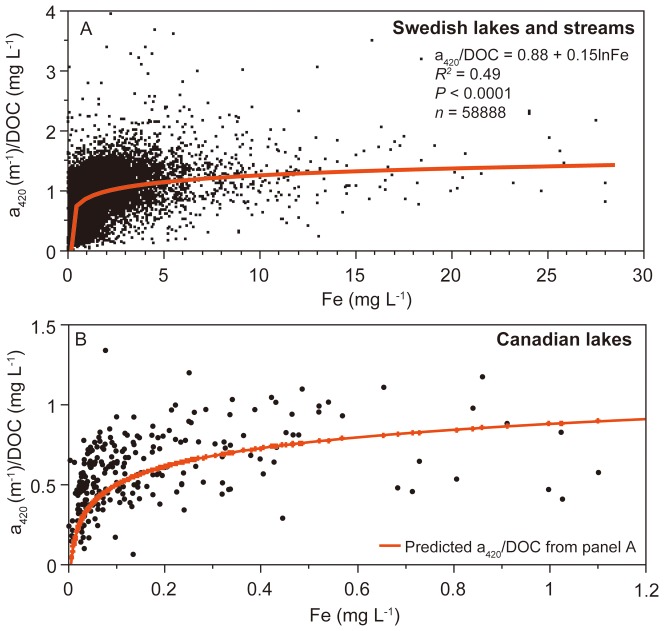
Relationships between iron (Fe) and the carbon specific absorbance (a_420_/DOC) based on all available data from Swedish lakes, streams and river mouths (panel A) and confirmed by data from Canadian lakes (panel B). In panel B we predicted a_420_/DOC by using the regression equation of panel A and obtained the regression line which is shown. Note the different scales between panel A and B.

Although DOC and a_420_ co-varied well we observed large variations in a_420_/DOC between 58888 Swedish water samples, ranging from 0.22 (5^th^ percentile) to 1.12 (95^th^ percentile) with a median of 0.69. Variations in a_420_/DOC were best explained by Fe, pH and Si (highest VIP values, all exceeding 1.8, in a partial least squares analysis using 38 water and catchment variables from 5837 waters as input variables). Taking all available 58888 water samples into consideration Fe showed a positive relationship to a_420_/DOC. The relationship was logarithmic, with fastest changes in a_420_/DOC at Fe concentrations below 1 mg L^−1^ (Fig. 2a). At Fe concentrations >5 mg L^−1^ a_420_ and DOC approached a 1:1 ratio (Fig. 2a). The equation of the logarithmic relationship between Fe and a_420_/DOC from the Swedish lakes was also valid for a set of Canadian lakes (Fig. 2b).

### Fe and DOC along the aquatic continuum and effects on a_420_

**Figure 3:**
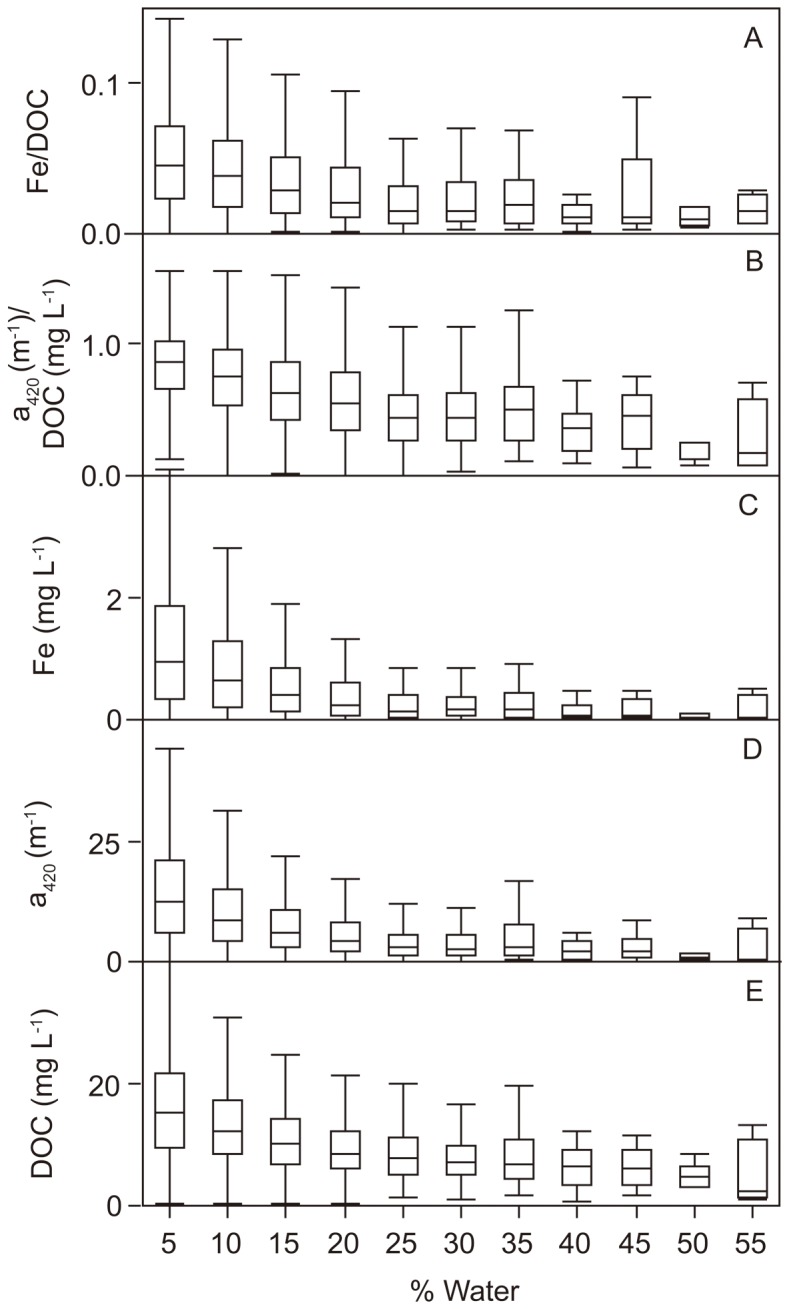
Iron (Fe), dissolved organic carbon (DOC), absorbance (a_420_), Fe/DOC and a_420_/DOC ratios in relation to the percentage of lake surface area in the catchment area (% Water). For the figure, site-specific long-term median data of 5837 different lakes and streams were used. Taking the median of each of the 11% Water categories and applying a simple exponential decay along the % Water gradient we received highly significant results (*P*<0.0001, *n* = 11; *R^2^* = 0.78 for Fe/DOC in panel A, *R^2^* = 0.84 for a_420_/DOC in panel B, *R^2^* = 0.95 for Fe in panel C, *R^2^* = 0.89 for a_420_ in panel D and *R^2^* = 0.88 for DOC in panel E).

**Figure 4:**
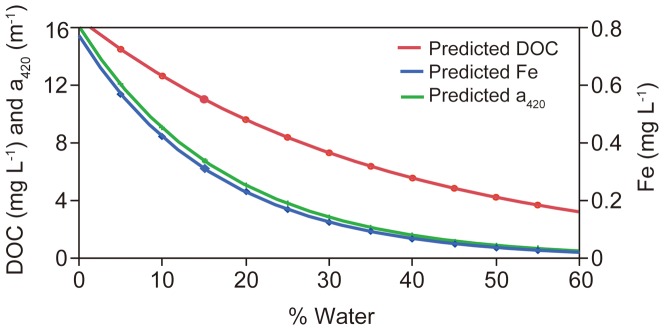
Decreasing iron (Fe), dissolved organic carbon (DOC) and absorbance (a_420_) with increasing percentage of lake surface area in the catchment area (% Water). The figure shows the predicted values of Fe, DOC and a_420_ from the simple exponential decay functions along the % Water gradient presented in Fig. 3. Fe and a_420_ decline equally fast with increasing % Water. The Fe and a_420_ decline is substantially faster than the decline of DOC. % Water can be seen as a proxy for water retention in the landscape (see methods).

Fe and DOC concentrations co-varied generally well, both in Swedish and Canadian waters (linear relationship on log-transformed data: *R^2^* = 0.44, *P*<0.0001, *n* = 58888 and *R^2^* = 0.58, *P*<0.0001, *n = *247, respectively). The co-variation was strongest within and between small headwater streams (linear relationship on log-transformed data: *R^2^* = 0.65, *P*<0.0001, *n* = 1421), and weakest within and between river mouth waters (linear relationship on log-transformed data: *R^2^* = 0.27, *P*<0.0001, *n* = 11667). The slopes between the Fe-DOC relationships varied substantially along the aquatic continuum, also reflected by significant differences in Fe/DOC ratios between lakes and running waters (Wilcoxon-test: *P*<0.0001). Median Fe/DOC ratios in headwater streams, non-headwater streams and river mouths had a value of 0.05 while the median value for lakes was 0.03 and for large lakes as low as 0.01. Thus, lakes contained less Fe in proportion to DOC compared to running waters. Also absolute Fe concentrations were lower in lakes, and we found significantly lower Fe concentrations in lakes and large lakes compared to headwater streams, non-headwater streams and river mouths (Wilcoxon-each-pair-test: *P*<0.0001). Along with lower Fe in lakes also a_420_ was at low levels. The DOC-specific absorbance, i.e. a_420_/DOC, showed similar patterns as Fe/DOC: highest in streams and lowest in lakes, in particular large lakes. Relating Fe, DOC and a_420_ to the percentage of lake surface cover in the catchment area we found a highly significant negative relationship (Fig. 3). The Fe and a_420_ decrease with increasing % Water was faster than for DOC, resulting in significantly decreasing a_420_/DOC and Fe/DOC ratios with increasing % Water (Fig. 3). The decreases in Fe and a_420_ along the % Water were proportionally similar in size (Fig. 4).

### Fe, DOC and a_420_ temporal changes

**Figure 5:**
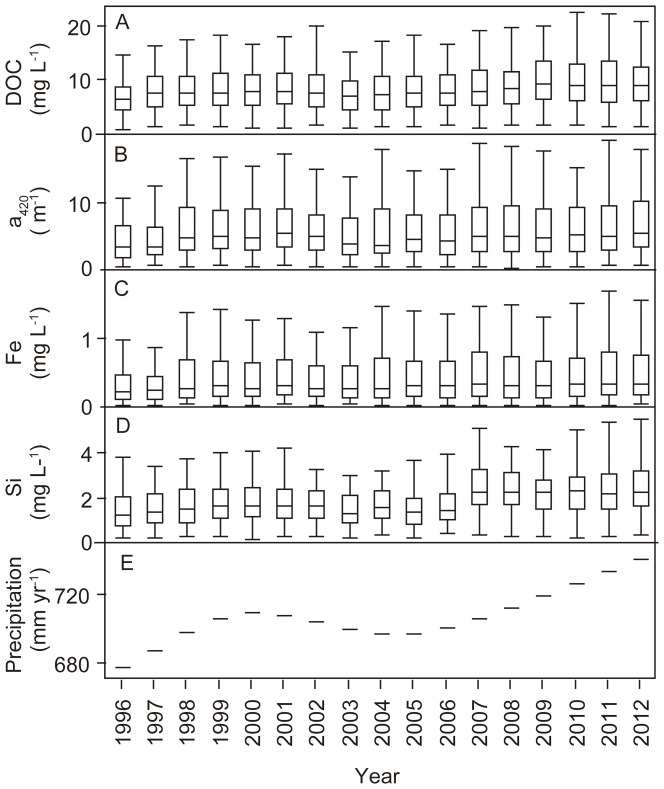
Temporal development of dissolved organic carbon (DOC), absorbance (a_420_), iron (Fe) and reactive silica (Si) in Swedish freshwaters and changes in long-term annual precipitation across Sweden since 1996. The DOC, a_420_, Fe and Si data (panels A–D) are based on annual mean values from 66 lakes, streams and river mouths for which complete monthly time series were available. Thus, for each year, 66 data points have been used for the percentile calculations. For panel E, 10-year running means of data from entire Sweden have been used (see methods).

Analyzing time series of Fe, DOC and a_420_ from 66 water (34 river mouths, 21 lakes and 11 streams) since 1996 we found significantly increasing trends for DOC in 43 waters, for Fe in 29 waters and for a_420_ in 25 waters (Mann-Kendall: *P*<0.05; Fig. 5). None of the water systems showed significantly decreasing trends in Fe, DOC and a_420_. In 13 waters we observed significantly increasing trends in Fe/DOC (Mann-Kendall: *P*<0.05). In 8 of these waters also a_420_ significantly increased (Mann-Kendall: *P*<0.05). Significant trends in a_420_/DOC were rare for this time period, only occurring in 6 waters (Mann-Kendall: *P*<0.05). Apart from Fe, DOC and a_420_ trends, we found significantly increasing trends also for the variables that were most important for a_420_ variations: Si showed significantly increasing trends in 43 waters (Fig. 5), pH in 10 waters, and a_420unfiltered_/a_420filtered_ in 10 waters (Mann-Kendall: *P*<0.05). At the same time as Fe, DOC and a_420_ increased in Swedish freshwaters, also Sweden-specific long-term running means of precipitation and growing season length significantly increased during 1996 to 2012 (Mann-Kendall: *P*<0.001; Fig. 5). There was no significant trend in long-term running means of air temperatures across Sweden during this time period (Mann-Kendall: *P*>0.05).

## Discussion

### Additive Fe effects on a_420_

As shown in earlier studies (e.g. [20]–[22]), our analyses of more than 58000 water samples from the Swedish and Canadian boreal and hemiboreal region confirmed that a_420_ co-varied strongest with DOC concentrations, suggesting that DOC is the overall main driver for water color in boreal waters. Although strong, the DOC-a_420_ relationship still exhibited considerable variation across temporal and spatial scales. We attribute the variability in a_420_/DOC ratios to an additive effect of Fe on a_420_, as indicated by a substantial model improvement with inclusion of Fe as independent variable.

An additive Fe effect on absorbance, apart from a DOC effect, has earlier been related to Fe-DOC complexation where highest absorbance was reached when Fe was bound to DOC [10], [23]. The strong co-variation between Fe and DOC in our waters, in particular in headwaters, suggests that Fe-DOC complexation is ubiquitous. Laboratory studies have shown that Fe-DOC complexation will reach a plateau when dissolved organic matter is saturated with Fe, with no further Fe-DOC binding and no further increases in carbon specific absorbance occurring when additional Fe is added [11]. Using waters from a humic lake in the laboratory, [10] found that Fe and carbon specific absorbance showed a strong positive relationship up to 1 mg L^−1^ Fe. At higher Fe concentrations, increases in carbon specific absorbance became small. [10] used a quadratic function to describe the relationship between Fe and carbon specific absorbance. We used a here a logarithmic function since we suggest that a_420_/DOC reaches a constant value along a Fe concentration gradient when DOC is saturated with Fe. The threshold value of 1 mg L^−1^ Fe as a breakpoint for a substantial additive effect of Fe (apart from the DOC effect) on absorbance, seems to hold even for boreal waters in general as we found relatively constant a_420_/DOC ratios when Fe exceeded 1 mg L^−1^ (Fig. 2a and b). Most of the boreal waters analyzed in this study had Fe concentrations less than 1 mg L^−1^, suggesting that Fe increases will result in a_420_/DOC increases in many freshwaters of the boreal and hemiboreal region.

Provided that variations in a_420_/DOC are influenced by Fe-DOC complexation with highest ratios when Fe is bound to DOC, we expect pH to play a major role for a_420_/DOC variations due to a strong pH effect on Fe complexation [8]. This expectation was supported by a significant pH effect on a_420_/DOC in our PLS models. Based on our results we suggest that water color is primarily driven by DOC but that Fe when it is bound to DOC will cause additional browning of waters.

### Lakes as Fe sinks and effects on a_420_

When Fe and DOC are imported from soils into waters they are usually tightly coupled, in this study indicated by highly significant Fe and DOC relationships in headwaters (*R^2^* = 0.65, *P*<0.0001). In headwaters Fe, DOC and a_420_ reached maximum values while they all declined with increasing % Water in the catchment (Fig. 3), which we use here as a proxy for water retention in the landscape (see methods). We suggest that decreases in Fe, DOC and a_420_ along the aquatic continuum are a result of several Fe and DOC transformation processes during transport from land to sea: phototransformation [24], [25], flocculation and burial in lake sediments [15], microbial degradation [26] and dilution by inputs of waters from other sources that frequently enter downstream systems [12] and that might be poor in DOC and Fe. We found that lakes were particularly efficient at removing Fe and a_420_, resulting in decreased Fe/DOC and a_420_/DOC ratios. Since the decline in Fe and a_420_ in relation to DOC along the % Water gradient was proportionally equal in size we suggest that an a_420_ decrease during water transport through the landscape is a result of a Fe loss in lakes. Fe in lakes can be lost by flocculation and sedimentation processes which has been shown to affect a_420_ [15]. Fe flocculation and sedimentation processes can result in a selective Fe loss in comparison to DOC as recently reported and discussed for the large Swedish lake Mälaren [7]. That sedimentation of Fe complexes takes places in lakes finds also evidence in a recent study by [27] who frequently detected Fe-OC complexes in all kinds of sediments around the globe. We suggest that Fe-DOC complexes that have been exported from soils are lost along the aquatic continuum. This suggestion is supported by a study of [28] who found that in Swedish river mouths waters up to 99% of dissolved Fe occurred as ferrihydrite which is rarely bound to organic matter [29]. Less frequent occurrence of Fe-DOC complexes in river mouths waters might explain why our Fe-DOC relationships became weaker along the aquatic continuum.

Decreases in a_420_/DOC along the aquatic continuum have been observed earlier [13]. It was suggested that the selective a_420_ loss in comparison to DOC is a result of DOC transformation processes during transport from land to sea. Here we relate the a_420_/DOC decline along the aquatic continuum for the first time to Fe losses in lakes. In [7] it was shown that in the large lake Mälaren Fe decreased 6.3 times faster than imported DOC. We found here that Fe decreased on average only 2.3 times faster than DOC along a water retention time gradient (Fig. 3). This discrepancy may result from the fact that water retention of a single lake ecosystem is not comparable to water retention in the landscape [12], in particular not since we use here % Water as a proxy for water retention in the landscape. In addition, most of our study lakes were small unproductive shallow lakes with frequent events of sediment resuspension [30] where the burial capacity of Fe might be limited.

### Increasing Fe contribution to a_420_ on a temporal scale

**Figure 6:**
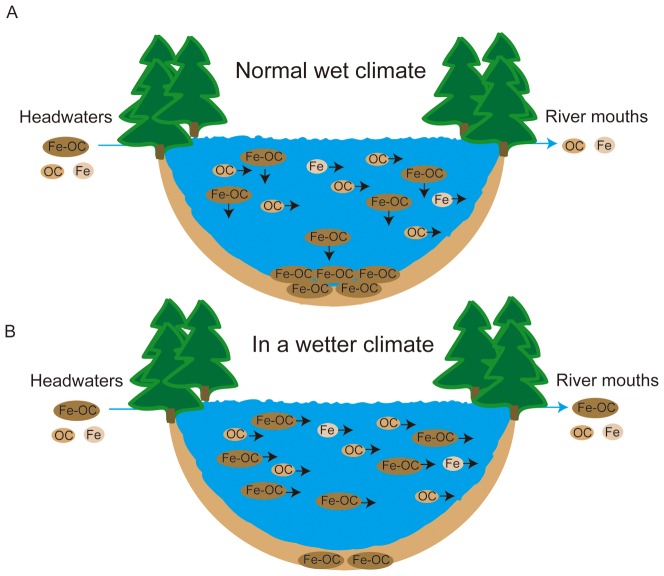
Fate of iron (Fe), dissolved organic carbon (DOC) and absorbance (a_420_) along the aquatic continuum under normal wet conditions (panel A) and in a wetter climate (panel B). When water travels from headwaters to river mouths and passes lakes Fe, DOC and a_420_ all decline (compare Fig. 3 and Fig. 4). It is suggested that a concomitant Fe, DOC and a_420_ decline in surface waters of lakes is a result of Fe-OC complexes that can flocculate and reach bottom waters and sediments (panel A). In a wetter climate with a consequent faster flushing of waters through lakes the settling of Fe-OC complexes towards bottom waters and sediments becomes less efficient and Fe-OC complexes reach downstream waters, where they cause strong declines in a_420_ (panel B). The conceptual figure assumes that Fe-OC complexes mainly originate from soils.

Like [6] we found significant increases in DOC, Fe and a_420_. [6] proposed a number of explanations why Fe concentrations and thereby a_420_ in Swedish waters might increase, one being climate change induced increases in anoxic conditions in organic soils. Changes of processes along the aquatic continuum were not a focus of their study and consequently not taken into consideration. We propose here that changes in Fe and consequently in a_420_ might indeed be related to increasing Fe soil exports but that changes in the Fe processing along the aquatic continuum also need attention. We found that Fe and a_420_ losses along the aquatic continuum decrease with decreasing water retention in the landscape (Fig. 4). Especially lakes played a central role for Fe and a_420_ losses along the aquatic continuum. We suggest that faster water flushing through lakes due to increased precipitation as observed across Sweden (Fig. 5) will result in higher Fe and a_420_ in downstream water systems compared to normal wet years (Fig. 6). According to our study, lakes are efficient in removing Fe from the water column. Thus, lakes function not only as efficient DOC sinks [30] but probably also as efficient Fe sinks which has effects on a_420_, in particular on a_420_/DOC. If Fe is lost in lakes in form of Fe-DOC complexes then faster water flushing through the landscape would imply less time for sedimentation of Fe-DOC complexes in lakes with consequent more Fe-DOC complexes reaching the sea (Fig. 6).

From our observed widespread increasing DOC trends across Swedish freshwaters we expected more significant a_420_ increases in the waters. According to our results a_420_ increases were mainly restricted to waters in which also Fe increased. Significant DOC trends that obviously did not affect a_420_ might be a result of DOC quality changes, in particular a change towards less colored DOC. DOC becomes less colored towards deeper soil layers where DOC is more extensively processed [31]. These deeper soil layers are reached by groundwater. Since we found significant increases in Si which is a mineralization product and an indicator of groundwater inputs [32] we attribute increases in less colored DOC to an increased export of DOC from deeper soil layers, probably from mineral soil layers that are rich in both Si and Fe. The increase in DOC exports from deeper soil layers might be caused by the increase in long-term precipitation (Fig. 5). The significance of Si on a_420_ was not only detectable on a temporal scale but also on a spatial scale: using PLS, Si was one of the most important variables explaining a_420_. Thus, understanding a_420_ changes requires an understanding of DOC sources that determine the proportions between colored and uncolored DOC.

In case less colored DOC presently increases as suggested above, we attribute a_420_/DOC increases to Fe concentration increases. As discussed above climate change induced decreases in the efficiency of Fe transformation processes along the aquatic continuum might be one explanation for Fe concentration increases but increased Fe soil exports are also highly likely. We argue, like we did for DOC quality, that due to a long-term precipitation increase and a longer growing season length deeper soil layers that are usually rich in Fe and Si are drained. This argumentation corresponds to the results of [33] who reported on increased Fe exports from soils with increasing wetness. Since we also found a significant increase in particulate matter, i.e. in a_420unfiltered_/a_420filtered_, we suggest that water flushing through soils has increased. We further suggest that podzolization plays an important role for the fate of Fe. Podzolization is a process where Fe is complexed with dissolved organic matter and transported downward in the soil profile [34]. If these deeper soil profiles are flushed Fe-DOC complexes will be exported from soils into surface waters. Podzolization is especially strong beneath conifers [34], which might explain why the percentage of coniferous forest in the catchment area came out as the most important catchment variable in our a_420_ model.

Finally, Fe increases might also be a result of mineralization rate increases as a response to the longer growing seasons. Like runoff changes, mineralization rate changes would also result in concomitant Fe and Si changes, as we observed in this study.

We conclude that DOC and Fe soil exports as well as DOC and Fe transformation processes during transport from land to sea need to be considered to understand why many freshwaters are browning. Brownish waters influence a number of ecosystem services including biodiversity [35], fish production [36], and drinking water quality [37]. Future studies on proportions between colored and uncolored DOC and between dissolved monomeric inorganic and colloidal Fe are strongly needed. First when these proportions have been quantified we will be able to fully understand how water color will respond to further environmental changes.
