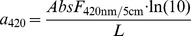# Correction: Browning of Boreal Freshwaters Coupled to Carbon-Iron Interactions along the Aquatic Continuum

**DOI:** 10.1371/journal.pone.0106357

**Published:** 2014-08-19

**Authors:** The PLOS ONE Staff

In the “Variables” section of the Materials and Methods, Equation 1 is represented incorrectly. Please see the corrected equation below.